# Early-Onset Colon Cancer: A Narrative Review of Its Pathogenesis, Clinical Presentation, Treatment, and Prognosis

**DOI:** 10.7759/cureus.45404

**Published:** 2023-09-17

**Authors:** Elvina C Lingas

**Affiliations:** 1 Hospital Medicine, New York University (NYU) Langone Health Long Island Community Hospital, Patchogue, USA

**Keywords:** early-onset colon cancer, cancer immunotherapy, young-onset colon cancer, colon cancer prevention, cancer pathogenesis, cancer therapy, lynch syndrome phenotype, familial adenomatosis polyposis, early onset, colorectal cancer

## Abstract

Colon cancer remains a leading cause of cancer-related deaths, and there has been a rise in the incidence of early-onset colon cancer or colon cancer diagnosed before the age of 50 years old. Early-onset colon cancer has several differences in clinical presentation, as well as histopathology, genetic alteration, and molecular profiling. Early-onset colon cancer can be differentiated into familial type that includes hereditary familial syndrome and sporadic type. Demographic variance also exists in both developing and developed countries. Due to the rising incidence of colon cancer diagnosed in younger age, it is imperative to examine the available evidence regarding the mortality rate of early-onset colon cancer. Colon cancer is affected by numerous modifiable and non-modifiable risk factors. Increasing obesity and lifestyle disorders in the younger population, such as smoking, may influence this increasing trend. There are existing guidelines for colon cancer screening in both average-risk and high-risk individuals. This narrative review aims to highlight the pathogenesis of early-onset CRC; its clinical presentation, treatment, prognosis; and how it differs from late-onset CRC.

## Introduction and background

Colorectal cancer (CRC) usually presents with nonspecific symptoms, such as blood in stools, altered bowel habits, fatigue, and weight loss [1]. There is an increasing concern regarding the rise of CRC in younger populations. Epidemiology data from different countries showed increasing incidence [2]. Increasing obesity and unhealthy lifestyles, such as Westernized high-fat diets and smoking, may have influenced this trend [3,4]. Screening colonoscopy and removal of precancerous polyps are shown to decrease mortality [5-7].

A proportion (10%) of newly diagnosed CRC cases are diagnosed before the age of 50, making it an emerging health problem [8]. The five-year survival rate for CRC is approximately 60% for the localized stage but declines to 14% for distant metastasis [9]. Some studies have shown that early-onset colon cancer has a lower survival rate compared to late-onset colon cancer, signifying potential molecular biology difference [10]. Early-onset CRC has been shown to have increased microsatellite instability [11], higher frequency of gene mutation [12], and often more poorly and undifferentiated cancer [13]. The characteristics and molecular pathogenesis of early-onset colon cancer differ from those of late-onset colon cancer, as it has a wide spectrum of diseases [14]. Early-onset CRC also is often delayed in diagnosis [15], which could be related to clinicians not having this diagnosis high in their differentials with the non-specific symptoms. There is no specific treatment for early-onset CRC. The treatment protocol is based on staging as late-onset CRC [16].

In this review, we discuss the incidence, pathogenesis, risk factors, clinical presentation, treatment, and prognosis of early-onset colon cancer.

This article was previously posted to the Authorea preprint server on June 12, 2023.

## Review

Incidence and epidemiology

Recent studies have demonstrated an increased number of colon cancer cases diagnosed in populations younger than 50 years of age. Despite advances in treatment and screening modalities, CRC remains the third most common cancer and second most common cause of cancer-related deaths worldwide [17]. There is a rising incidence of CRC in populations younger than 50 years of age in high-income countries, such as Australia, Canada, the United States, Denmark, New Zealand, and the UK [2]. It accounts for 10% of newly diagnosed colon cancer cases and contributes to the overall mortality [18]. In Western countries, such as the United States, 5% of all CRC-diagnosed patients are <45 years of age based on the Surveillance, Epidemiology, and End Results Program (SEER) [8]. Eastern European countries reported an annual increase of 7.4% in colon cancer among young individuals between 2008 and 2016 [19]. The rising cases of early-onset colon cancer are also seen in other countries, such as Egypt and Iran [5]. It is evident that an increasing incidence of early-onset CRC is observed in both developed and developing countries, and there is also decreasing mortality in developed countries, such as Japan, the United States, Australia, and Western European countries, which is likely due to the advancement of treatments and screening modalities.

Meanwhile, there is increasing mortality in countries, such as Mexico, Brazil, and Eastern European countries, which could be related to health inequality due to a lack of resources [20]. From 2008 to 2012, age-standardized incidence of early-onset CRC was 12.9 per 100,000 in Korea, which is the highest in Asia, followed by Japan (9.7), the Philippines (6.5), China (6.4), and India (3.5) [2]. It is important to note that Asia accounts for half of the colon cancer burden worldwide, especially China, which has the highest deaths and disability rates from colon cancer, attributable to dietary risks, followed by the United States, India, and Japan [21,22]. A similar contrasting result was documented in this sub-Saharan Africa study, where crude incidence was reported to be low (4.40 per 100,000) [23]; however, it is associated with higher mortality and morbidity [24]. This racial disparity is reflected in this study using National Cancer Institute (NCI) epidemiology data [25].

Pathogenesis

Role of Genetics and Molecular Profile

Compared to CRC diagnosed in early adults, early-onset CRC has some distinctive pathological features, and current knowledge has postulated that it is a heterogenous disease with both sporadic and familial cases, in which the exact molecular cause of this phenomenon is yet to be clarified [11]. Studies have shown that there is a difference in genetic variants between early- and late-onset CRC. In a 2021 Spanish Ministry-funded study that examined both familial and sporadic early-onset CRC, approximately 13% of patients with early-onset CRC were found to carry pathogenic germline variants in known cancer predisposition genes, and around 2.5% of cases harbor genes that are not associated with colorectal cancer, such as *BRCA1*, *BRCA2*, *TP53*, *ATM*, *CHEK2*, *PALB2*, and *CDKN2A* [26]. 

Patients with early-onset colon cancer tend to have more hereditary syndromes (e.g., familial adenomatous polyposis, MUTYH-associated polyposis, Lynch syndrome, and certain hamartomatous polyposis conditions) [27]. Lynch syndrome accounts for <5% of the total CRC cases and one-third of early-onset CRC cases, while familial adenomatous polyposis (FAP) only accounted for less than 1% of all CRC cases [28-29]. Sporadic early-onset CRC are classified into chromosomal instability (CIN) and microsatellite instability (MSI) [29]. There is evidence that MSI tumors are different from microsatellite stability (MSS) tumors, such as higher frequency of BRAF mutations and a lower frequency of *KRAS*, *APC*, and *TP53* mutations [30].

Molecular profiling studies have shown that patients with early-onset CRC have distinct molecular subtypes compared to older patients, including increased expression of *CMS1* (consensus molecular subtype) of unclear etiology and increased MSI [7]. In the two clinical trials FIRE3 and LUME-Colon-1, patients with late-onset CRC have mostly *CMS2* and *CMS4* expressions, and it is worth noting that *CMS1* was associated with poorer survival rate, while *CMS2* and *CMS4* were associated with better survival rates [31-32].

Epigenetic alterations, such as changes in DNA methylation patterns, are observed in early-onset CRC. Hypomethylation of long interspersed nuclear elements is significantly higher in younger patients than in older patients and is associated with a lower survival rate [33,34]. It is thought that early-onset CRC has an MSI expression from the methylated *MLH1* gene that is responsible for controlling DNA replication, which is also controlled by other genes, such as *MSH2* and *MSH6*. This process led to mutations in the *BRAF* gene, and the *BRAF* V600E mutation is widely accepted as a prognostic factor of sporadic CRC with MSI [35].

There are however reports that showed MSS-type tumors as a majority finding in early-onset CRC, lacking DNA repair capabilities [36]. A population-based study only documented 17% of MSI in early-onset CRC [37]. Another role identified is the methylation of CpG islands. High levels of CpG island methylator phenotype (CIMP) are associated with poor differentiation and MSI and BRAF mutations [12]. Perea et al. showed that young-onset CIMP-high CRCs were associated with *MMR* gene germline mutations. By contrast, late-onset CIMP-high CRCs were more likely to be sporadic MSI tumors [36]. *MMR* may have some therapeutic implications and may serve as targets for immune inhibitors [38,39]. *LINE-1 *gene hypomethylation’s expression in both late- and early-onset CRC was compared by Antelo et al. and was shown to have lower level in the first group [33].

*LINE-1* hypomethylation is associated with CIN [40], but its value in prognostication is yet to be elucidated. Genetic testing should be recommended for young patients with a history of early-onset colorectal cancer in the family [12,41].

**Figure 1:**
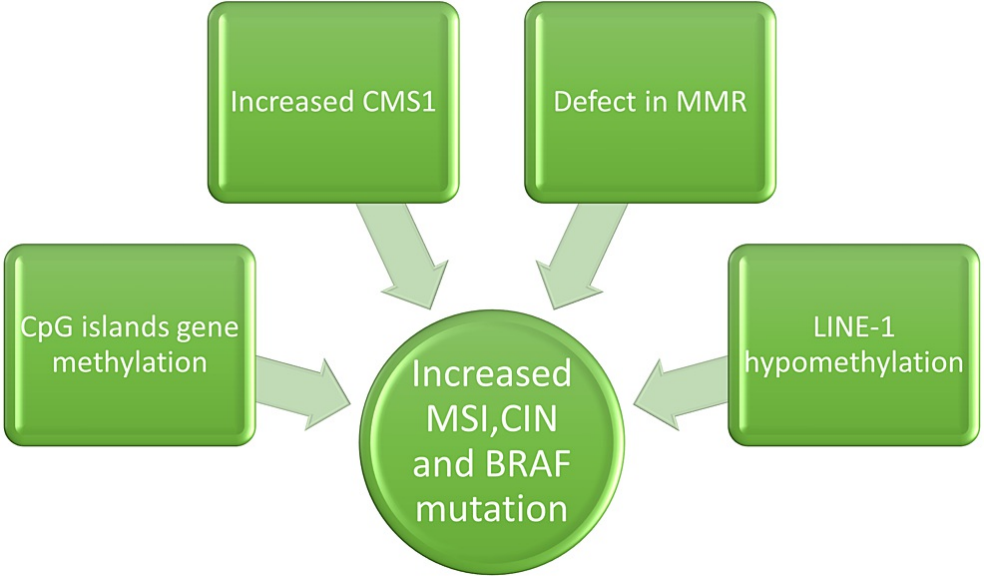
Schematic representation of the molecular profile in early-onset colon cancer CMS1: consensus molecular subtype 1, MSI: microsatellite instability, CIN: chromosomal instability, MMR: mismatch repair Credits: Author (Elvina Lingas)

Histopathology features show that early-onset CRC often has signet ring cell histology, lymphovascular invasion, and perineural invasion compared with late-onset CRC. These features are associated with aggressive tumors and a worse prognosis [13]. The molecular profile in early-onset colon cancer is illustrated in Figure 1. 

Lifestyle and Modifiable Risk Factors

**Figure 2:**
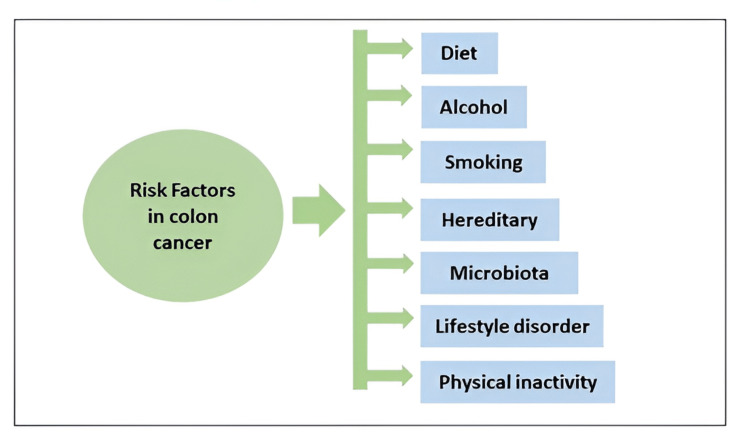
Schematic representation of the risk factors involved in the initiation and progression of colon cancer Credits: Author (Elvina Lingas)

While genetics play an important role, lifestyle and environmental factors play an even bigger role in the pathogenesis of early-onset CRC [3,4]. Various risk factors of CRC are illustrated in Figure 2.

Highly processed diet with high intake of red meat and low fiber intake has been associated with increased risk of colon cancer [42,43]. Deep-fried food interferes with lipid metabolism, increases oxidative stress, and increases carcinogen production [44]. Food high in dietary trans fat is also associated with an increased risk of CRC [44]. Habitual red meat consumers have 20% higher risk of developing CRC when compared to occasional consumers [45].

Regular alcohol consumption is associated with colon cancer with an increased risk parallel to the duration of alcohol use [46]. Moderate to heavy alcohol consumption has a 20% additional risk of developing CRC [46]. Alcohol metabolism increases oxidative stress, disturbs the colon epithelial barrier and the colon microbiome, and produces carcinogens [47]. Studies have shown that the microbial environment of the colon, the concentration of bile salts, metabolites, and the level of oxygen also play a role in early-onset colon cancer [48].

Cigarette smoking increases the risk of developing colon cancer [49], increases the expression of 5-LOX in colon cancer, disturbs the apoptosis mechanism, and upregulates nicotinic acetylcholine receptors (nAChRs) [50]. These receptors are responsible for multiple oncogenic signaling pathways and promote tumor development and progression [51].

Physical activity decreases the risk CRC development [52]. Sedentary lifestyle sitting for more than 14 hours per week may increase the risk of developing early-onset CRC [53]. Colon cancer survivors with high levels of physical activity have a lower risk of recurrence [54].

The rising trend of obesity in the young population is worrisome especially because studies have shown that it may increase the risk of developing CRC and may contribute to the increasing incidence of early-onset CRC [3]. Carcinogenic mechanism of obesity could stem from insulin resistance, which increases chronic inflammation, oxidative stress, DNA damage, and insulin-like growth factor-1 (IGF-1) levels, further stimulating cell proliferation [55]. Obese individuals with type 2 diabetes mellitus has increased risk of developing colon cancer, which is thought due to prolonged exposure to high levels of insulin in the colon [56]. Weight loss seems to help in lowering this risk [57].

Clinical Presentation, Diagnosis, and Staging

Most patients with colon cancer have nonspecific symptoms, such as fatigue due to anemia or altered bowel habits [58]. A thorough medical history and physical examination are warranted, including a routine complete blood count, which may show anemia, a complete chemistry profile including a liver function test, and occasionally a fecal occult blood test or fecal immunochemical test [58,59]. Different from late-onset CRC, early-onset CRC often present with bowel obstruction [60]. The gold standard of diagnostic studies is screening colonoscopy to check for polyps. Tissue diagnosis is obtained via biopsy. Further molecular testing is performed to detect gene variants, such as *KRAS*, *NRAS*, and *BRAF* genes, as well as *HER2* protein and *NTRK* genes [61]. MSI testing is performed to detect high levels of gene changes and detect mismatch repair (MMR) genes to discover genes, such as *MLH1*, *MSH2*, *MSH6*, *PMS2*, and *EPCAM*, which may offer information regarding prognosis [62]. Computed tomography (CT) scan may show a mass or metastasis to surrounding organs, which can sometimes be the first sign, especially if the patients are present in later stages [63].

In 2018, the American Cancer Society (ACS) updated its screening guideline to recommend starting colon cancer screening starting at age 45 years for average-risk individuals by either high-sensitivity stool-based test or a structural (visual) examination based on preference and availability. Any positive testing of noncolonoscopy screening testing needs to be confirmed with colonoscopy in a timely manner [64]. There is a variation in screening guideline worldwide, with countries, such as Canada and the UK, recommending initiating screening at age 50 [65,66], while Japan recommended to start screening with annual fecal immunochemistry testing (FIT) at age 40 years [67]. Patients with hereditary and familial syndrome, such as Lynch syndrome and FAP, are required to have expedited colon cancer screening in additional to other cancer screening, such as esophagogastroduodenoscopy (EGD) [27].

Studies have documented a major delay in diagnosing early-onset CRC, ranging from a few months delay [68,69] to two years in some case report [15]. This is potentially due to the fact that the symptoms are often non-specific and clinicians do not have high index of suspicion of colon cancer in this population [70]. This delay may contribute to advanced stages upon presentation [71] as several studies have documented that early-onset CRC patients often present with stage III or even stage IV upon diagnosis [2,60,72-74]. A recent multicenter retrospective study in Korea by Son et al. showed that young patients with CRC present with more undifferentiated or poorly differentiated carcinoma and a higher rate of perineural invasion and hence are more likely to receive adjuvant chemotherapy and multidrug agents. Interestingly, they have better recurrent free survival (RFS) compared to older patients possibly due to better compliance with chemotherapy [75].

**Figure 3:**
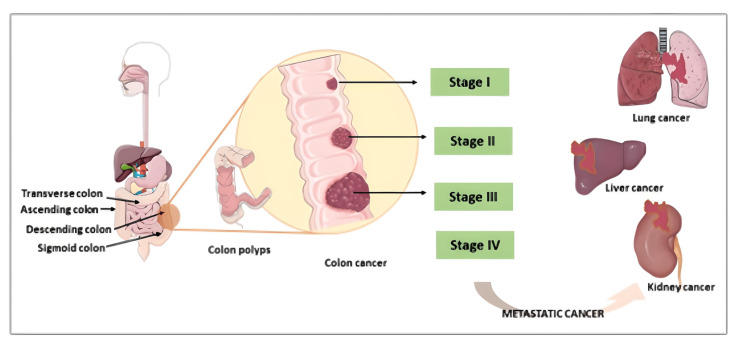
Schematic representation of colon cancer and its various stages Credits: Author (Elvina Lingas)

The stages of cancer were determined based on the extent of tumor extension and involvement of distant organs (Figure 2). Colon cancer staging system uses the tumor/node/metastasis (TNM) classification system by the American Joint Committee on Cancer (AJCC) that are assigned by the characteristics of primary tumor (T), the extent of regional lymph node involvement (N), and distant metastasis (M). In addition, metastasis may be defined by the preoperative clinical assessment or pathological evaluation of metastatic tissues [76,77]. The development of colon cancer and its various stages is illustrated in Figure 3. The staging classification system based on the TNM classification system is shown in Table 1 and Table 2.

**Table 1:** TNM classification for colon cancer, including primary tumor (T), regional lymph node involvement (N), and distant metastasis (M) Credits: Author (Elvina Lingas), adapted from [72,73]

Tx	Primary tumor cannot be assessed
T0	No evidence of primary tumor
Tis	Carcinoma in situ: intraepithelial or intramucosal carcinoma (involvement of lamina propria with no extension through the muscularis mucosa)
T1	Tumor invades submucosa (through the muscularis mucosa but not into the muscularis propria)
T2	Tumor invades muscularis propria
T3	Tumor invades through the muscularis propria into the pericolorectal tissues
T4	Tumor invades the visceral peritoneum or invades or adheres to adjacent organ or structure
T4a	Tumor invades through the visceral peritoneum (including gross perforation of the bowel through tumor and continuous invasion of tumor through areas of inflammation to the surface of the visceral peritoneum)
T4b	Tumor directly invades or is adherent to other organs or structures

Nx	Regional lymph nodes cannot be assessed
N0	No regional lymph node metastasis
N1	Metastasis in one to three regional lymph nodes (tumor in lymph nodes measuring ≥0.2 mm) or any number of tumor deposits are present and all identifiable nodes are negative
N1a	Metastasis in one regional lymph node
N1b	Metastasis in two to three regional lymph nodes
N1c	Tumor deposit(s) in the subserosa, mesentery, or nonperitonealized, pericolic, or perirectal/mesorectal tissues without regional nodal metastasis
N2	Metastasis in four or more lymph nodes
N2a	Metastasis in four to six regional lymph nodes
N2b	Metastasis in seven or more regional lymph nodes

M category	M criteria
cM0	No distant metastasis by imaging or other studies, no evidence of tumor in distant sites or organs. (This category is not assigned by pathologists.)
cM1/pM1	Metastasis to one or more distant sites or organs or peritoneal metastasis is identified and/or microscopically confirmed.
cM1a/pM1a	Metastasis confined to one organ or site is identified without peritoneal metastasis and/or microscopically confirmed.
cM1b/pM1b	Metastasis to two or more sites or organs is identified without peritoneal metastasis and/or microscopically confirmed.
M1c/pM1c	Metastasis to the peritoneal surface alone or with other site or organ metastases and/or microscopically confirmed.

**Table 2:** Prognostic stage groups Credits: Author (Elvina Lingas), adapted from materials in [72,73]

Stage 0	Tis	N0	M0
Stage I	T1	N0	M0
	T2	N0	M0
Stage IIA	T3	N0	M0
Stage IIB	T4A	N0	M0
Stage IIC	T4B	N0	M0
Stage IIIA	T1-T2	N1/N1c	M0
	T1	N2a	M0
Stage IIIB	T3-T4a	N1/N1c	M0
	T2-T3	N2a	M0
	T1-T2	N2b	M0
Stage IIIC	T4a	N2a	M0
	T3-T4a	N2b	M0
	T4b	N1-N2	M0
Stage IVA	Any T	Any N	M1a
Stage IVB	Any T	Any N	M1b
Stage IVC	Any T	Any N	M1c

Treatment

Colon cancer treatment options consist of surgical and systemic treatments based on staging. Oncologic societies around the world, such as the European Society of Medical Oncology (ESMO), Japan Society of Medical Oncology (JSMO), Chinese Society of Clinical Oncology (CSS), Korean Association for Clinical Oncology, Malaysian Oncological Society (MOS), Singapore Society of Oncology (SSO), and Taiwan Oncology Society (TOS), have similar guidelines when it comes to treating both early- and late-onset colon cancer; however, more publications have proposed more aggressive surgical and nonsurgical modalities for early-onset CRC diagnosed in stages III and IV [78,79].

Surgery is the mainstay of treatment for localized diseases [66]. Patients who present with acute obstructive colon cancer often require a stent placement to relieve obstruction, and surgery is performed with subsequent stoma creation [80]. Laparoscopic-assisted colectomy is appropriate and curative in patients with suitable risks [81]. Neoadjuvant chemotherapy (induction chemotherapy before surgery) is often recommended for locally advanced tumors, and the systematic review by Zhou et al. showed a decrease in the number of affected lymph nodes, improved staging, and reduced post-operative morbidity compared to patients who did not receive chemotherapy while having an acceptable level of toxicity [82]. 

Some observational studies [83,84] have shown that adjunctive therapy, such as regular low doses of aspirin and other nonsteroidal anti-inflammatory drugs (NSAIDs), may have protective effects against colon cancer with tolerable side effects. The aspirin dose recommended for high-risk populations is 80-1200 mg/day depending on the stage and severity of disease; however, NSAIDs are not recommended for either general or high-risk populations considering their side effects [85]. A Swedish cohort study showed that hormonal therapy, such as estrogen alone or a combination of estrogen-progestin therapy, may reduce the risk of colon cancer mortality by 26% in women older than 40 years of age. This study did not specify the stage of colon cancer upon diagnosis and/or death [86].

Continuous advancements in precision oncology, including immunotherapy, are promising, including the clinical benefits of anti-EGFR treatments for metastatic colon cancer [87]. In the phase II REVERCE trial, the sequence of regorafenib (which is a tyrosine kinase inhibitor) followed by cetuximab is associated with a longer overall survival in metastatic colon cancer; however, of note, the result may not apply to the majority of colon cancer since the participants had unresectable colon adenocarcinoma with wild-type KRAS exon 2 after failure of fluoropyrimidine, oxaliplatin, and irinotecan [88]. CRYSTAL and OPUS randomized clinical trials demonstrated that adding cetuximab to standard FOLFIRI treatment (leucovorin, fluorouracil, irinotecan hydrochloride) in patients with KRAS wild-type metastatic colorectal cancer (mCRC) significantly improved the treatment outcome compared with chemotherapy alone [89,90].

Immunotherapy treatment modalities, such as PD-1 inhibitors (pembrolizumab and nivolumab) and CTLA-4 inhibitors (ipilimumab) offer promising results in the metastatic stage [91]. The phase II multicohort CheckMate 142 study confirms the long-term benefits of the ipilumab-nivolumab combination in metastatic colon cancer patients [92]. Ongoing and planned studies are zeroing on using circulating tumor DNA markers as prognostication factor and deciding on therapy escalation or de-escalation [93].

Prognosis

Early-onset colon cancer seems to have a poorer prognosis compared to older populations [94] and faster progression possibly due to different molecular and genetic variations, including increased gene expression associated with high-risk CMS subtype and chromosomal instability [31,32,95]. Early-onset colon cancer patients tend to have advanced stage upon diagnosis with higher mortality and are more likely to receive postoperative chemotherapy and multivalent regimens compared to older patients [96]. Some data suggest that the poor outcome of early-onset colon cancer is most likely due to the advanced stage of the disease [97].

Prognostication is based on the location of the tumor, depth of tumor invasion, tumor stage, tumor differentiation, surgery, pathological type, tumor size, lymph node metastasis, and distant metastasis [73]. The tumor site may have some prognostic value, but it requires further validation [98]. However, conflicting data do exist. A 2015 observation study using SEER data from 1988 to 2011 showed that younger patients have better survival [99]. This is also reflected in a similar observational study by Abdelsattar et al. [100] and in a multicenter cohort study by Son et al. This finding in particular was possibly due to the fact that younger patients tend to tolerate chemotherapy better and have better compliance to treatment [75]. The mortality rate seemed to be increased in early-onset CRC [101]. The survival data may be confounded by hereditary forms of colon cancer, which have better survival [102].

## Conclusions

Early-onset colon cancer has become an increasing major health burden. Aside from presenting more often with more aggressive histopathological features, studies have shown that they may have a different molecular profile compared to late-onset colon cancer. Sporadic-type early-onset colon cancer tends to have more chromosomal instability and microsatellite instability mutation. In some studies, early-onset colon cancer was found to have consensus molecular subtypes that reflected poor prognosis.

There is evidence of increasing unhealthy lifestyle in younger population. Smoking, physical inactivity, poor diet, and obesity can increase the risk of developing colon cancer. Increasing obesity and unhealthy lifestyle in younger population may contribute to the increasing incidence or early-onset colon cancer. There are conflicting data regarding colon cancer’s survival; however, there is sound evidence that younger patients tend to have more advanced stage upon diagnosis and delay in diagnosis. It is important for clinicians to recognize this and obtain a thorough examination for young patients presenting with non-specific gastrointestinal symptoms.

Colon cancer are staged by the TNM system. There is no specific treatment guideline or protocol for early-onset colon cancer compared to late-onset colon cancer. Immunotherapy has been implemented in colon cancer and shows promising results in several trials.

## References

[1] Mello MR, Moura SF, Muzi CD, GuimarÃes RM (2020). Clinical evaluation and pattern of symptoms in colorectal cancer patients. Arq Gastroenterol.

[2] Siegel RL, Torre LA, Soerjomataram I, Hayes RB, Bray F, Weber TK, Jemal A (2019). Global patterns and trends in colorectal cancer incidence in young adults. Gut.

[3] Stoffel EM, Murphy CC (2020). Epidemiology and mechanisms of the increasing incidence of colon and rectal cancers in young adults. Gastroenterology.

[4] Murphy CC, Singal AG, Baron JA, Sandler RS (2018). Decrease in incidence of young-onset colorectal cancer before recent increase. Gastroenterology.

[5] Mauri G, Sartore-Bianchi A, Russo AG, Marsoni S, Bardelli A, Siena S (2019). Early-onset colorectal cancer in young individuals. Mol Oncol.

[6] Castelló A, Amiano P, Fernández de Larrea N (2019). Low adherence to the western and high adherence to the mediterranean dietary patterns could prevent colorectal cancer. Eur J Nutr.

[7] Willauer AN, Liu Y, Pereira AA (2019). Clinical and molecular characterization of early-onset colorectal cancer. Cancer.

[8] Bray F, Ferlay J, Soerjomataram I, Siegel RL, Torre LA, Jemal A (2018). Global cancer statistics 2018: GLOBOCAN estimates of incidence and mortality worldwide for 36 cancers in 185 countries. CA Cancer J Clin.

[9] Siegel RL, Miller KD, Jemal A (2018). Cancer statistics, 2018. CA Cancer J Clin.

[10] Keum N, Giovannucci E (2019). Global burden of colorectal cancer: emerging trends, risk factors and prevention strategies. Nat Rev Gastroenterol Hepatol.

[11] Tezcan G, Tunca B, Ak S, Cecener G, Egeli U (2016). Molecular approach to genetic and epigenetic pathogenesis of early-onset colorectal cancer. World J Gastrointest Oncol.

[12] Silla IO, Rueda D, Rodríguez Y, García JL, de la Cruz Vigo F, Perea J (2014). Early-onset colorectal cancer: a separate subset of colorectal cancer. World J Gastroenterol.

[13] Johncilla M, Yantiss RK (2020). Histology of colorectal carcinoma: proven and purported prognostic factors. Surg Pathol Clin.

[14] Jeong MA, Kang HW (2019). Early-onset colorectal cancer [Article in Korean]. Korean J Gastroenterol.

[15] Myers EA, Feingold DL, Forde KA, Arnell T, Jang JH, Whelan RL (2013). Colorectal cancer in patients under 50 years of age: a retrospective analysis of two institutions' experience. World J Gastroenterol.

[16] Saraiva MR, Rosa I, Claro I (2023). Early-onset colorectal cancer: a review of current knowledge. World J Gastroenterol.

[17] (2020). Erratum: global cancer statistics 2018: GLOBOCAN estimates of incidence and mortality worldwide for 36 cancers in 185 countries. CA Cancer J Clin.

[18] Zaborowski AM, Abdile A, Adamina M (2021). Characteristics of early-onset vs late-onset colorectal cancer: a review. JAMA Surg.

[19] Wong MC, Huang J, Lok V, Wang J, Fung F, Ding H, Zheng ZJ (2021). Differences in incidence and mortality trends of colorectal cancer worldwide based on sex, age, and anatomic location. Clin Gastroenterol Hepatol.

[20] Center MM, Jemal A, Smith RA, Ward E (2009). Worldwide variations in colorectal cancer. CA Cancer J Clin.

[21] Allen L, Williams J, Townsend N, Mikkelsen B, Roberts N, Foster C, Wickramasinghe K (2017). Socioeconomic status and non-communicable disease behavioural risk factors in low-income and lower-middle-income countries: a systematic review. Lancet Glob Health.

[22] Deng Y, Wei B, Zhai Z (2021). Dietary risk-related colorectal cancer burden: estimates from 1990 to 2019. Front Nutr.

[23] Graham A, Adeloye D, Grant L, Theodoratou E, Campbell H (2012). Estimating the incidence of colorectal cancer in Sub-Saharan Africa: A systematic analysis. J Glob Health.

[24] Anugwom C, Braimoh G, Sultan A, Johnson WM, Debes JD, Mohammed A (2023). Epidemiology and genetics of early onset colorectal cancer-African overview with a focus on Ethiopia. Semin Oncol.

[25] Holowatyj AN, Ruterbusch JJ, Rozek LS, Cote ML, Stoffel EM (2016). Racial/ethnic disparities in survival among patients with young-onset colorectal cancer. J Clin Oncol.

[26] Daca Alvarez M, Quintana I, Terradas M, Mur P, Balaguer F, Valle L (2021). The inherited and familial component of early-onset colorectal cancer. Cells.

[27] Jasperson KW, Tuohy TM, Neklason DW, Burt RW (2010). Hereditary and familial colon cancer. Gastroenterology.

[28] Lynch HT, de la Chapelle A (2003). Hereditary colorectal cancer. N Engl J Med.

[29] Samowitz WS, Curtin K, Lin HH (2001). The colon cancer burden of genetically defined hereditary nonpolyposis colon cancer. Gastroenterology.

[30] Lanza G, Ferracin M, Gafà R (2007). mRNA/microRNA gene expression profile in microsatellite unstable colorectal cancer. Mol Cancer.

[31] Stintzing S, Wirapati P, Lenz HJ (2019). Consensus molecular subgroups (CMS) of colorectal cancer (CRC) and first-line efficacy of FOLFIRI plus cetuximab or bevacizumab in the FIRE3 (AIO KRK-0306) trial. Ann Oncol.

[32] Lenz HJ, Argiles G, Yoshino T (2021). Association of consensus molecular subtypes and molecular markers with clinical outcomes in patients with metastatic colorectal cancer: biomarker analyses from LUME-colon 1. Clin Colorectal Cancer.

[33] Antelo M, Balaguer F, Shia J (2012). A high degree of LINE-1 hypomethylation is a unique feature of early-onset colorectal cancer. PLoS One.

[34] Feinberg AP (2018). The key role of epigenetics in human disease prevention and mitigation. N Engl J Med.

[35] Loughrey MB, Waring PM, Tan A (2007). Incorporation of somatic BRAF mutation testing into an algorithm for the investigation of hereditary non-polyposis colorectal cancer. Fam Cancer.

[36] Perea J, Rueda D, Canal A (2014). Age at onset should be a major criterion for subclassification of colorectal cancer. J Mol Diagn.

[37] Salovaara R, Loukola A, Kristo P (2000). Population-based molecular detection of hereditary nonpolyposis colorectal cancer. J Clin Oncol.

[38] Popat S, Hubner R, Houlston RS (2005). Systematic review of microsatellite instability and colorectal cancer prognosis. J Clin Oncol.

[39] André T, Cohen R, Salem ME (2022). Immune checkpoint blockade therapy in patients with colorectal cancer harboring microsatellite instability/mismatch repair deficiency in 2022. Am Soc Clin Oncol Educ Book.

[40] Jones PA, Baylin SB (2007). The epigenomics of cancer. Cell.

[41] Gupta S, Provenzale D, Llor X (2019). NCCN guidelines insights: genetic/familial high-risk assessment: colorectal, version 2.2019. J Natl Compr Canc Netw.

[42] Mentella MC, Scaldaferri F, Ricci C, Gasbarrini A, Miggiano GA (2019). Cancer and Mediterranean diet: a review. Nutrients.

[43] Baena R, Salinas P (2015). Diet and colorectal cancer. Maturitas.

[44] Dhaka V, Gulia N, Ahlawat KS, Khatkar BS (2011). Trans fats-sources, health risks and alternative approach - a review. J Food Sci Technol.

[45] Campmans-Kuijpers MJ, Dijkstra G (2021). Food and food groups in inflammatory bowel disease (ibd): the design of the Groningen anti-inflammatory diet (GRAID). Nutrients.

[46] Jayasekara H, MacInnis RJ, Room R, English DR (2016). Long-term alcohol consumption and breast, upper aero-digestive tract and colorectal cancer risk: a systematic review and meta-analysis. Alcohol Alcohol.

[47] Rossi M, Jahanzaib Anwar M, Usman A, Keshavarzian A, Bishehsari F (2018). Colorectal cancer and alcohol consumption-populations to molecules. Cancers (Basel).

[48] Avril M, DePaolo RW (2021). "Driver-passenger" bacteria and their metabolites in the pathogenesis of colorectal cancer. Gut Microbes.

[49] Nam S, Choi YJ, Kim DW, Park EC, Kang JG (2019). Risk factors for colorectal cancer in Korea: a population-based retrospective cohort study. Ann Coloproctology.

[50] Zhao Y (2016). The oncogenic functions of nicotinic acetylcholine receptors. J Oncol.

[51] Hecht SS (2003). Tobacco carcinogens, their biomarkers and tobacco-induced cancer. Nat Rev Cancer.

[52] Leitzmann M, Powers H, Anderson AS (2015). European code against cancer 4th edition: physical activity and cancer. Cancer Epidemiol.

[53] Nguyen LH, Liu PH, Zheng X (2018). Sedentary behaviors, TV viewing time, and risk of young-onset colorectal cancer. JNCI Cancer Spectr.

[54] Ratjen I, Schafmayer C, di Giuseppe R (2017). Postdiagnostic physical activity, sleep duration, and TV watching and all-cause mortality among long-term colorectal cancer survivors: a prospective cohort study. BMC Cancer.

[55] Sádaba MC, Martín-Estal I, Puche JE, Castilla-Cortázar I (2016). Insulin-like growth factor 1 (IGF-1) therapy: mitochondrial dysfunction and diseases. Biochim Biophys Acta.

[56] Jurjus A, Eid A, Al Kattar S (2016). Inflammatory bowel disease, colorectal cancer and type 2 diabetes mellitus: the links. BBA Clin.

[57] Sinicrope FA (2022). Increasing incidence of early-onset colorectal cancer. N Engl J Med.

[58] Hillman RJ, Berry-Lawhorn JM, Ong JJ (2019). International Anal Neoplasia Society guidelines for the practice of digital anal rectal examination. J Low Genit Tract Dis.

[59] Young GP, Pedersen SK, Mansfield S (2016). A cross-sectional study comparing a blood test for methylated BCAT1 and IKZF1 tumor-derived DNA with CEA for detection of recurrent colorectal cancer. Cancer Med.

[60] Goldvaser H, Purim O, Kundel Y (2016). Colorectal cancer in young patients: is it a distinct clinical entity?. Int J Clin Oncol.

[61] Di Nicolantonio F, Vitiello PP, Marsoni S (2021). Precision oncology in metastatic colorectal cancer - from biology to medicine. Nat Rev Clin Oncol.

[62] Shia J (2021). The diversity of tumours with microsatellite instability: molecular mechanisms and impact upon microsatellite instability testing and mismatch repair protein immunohistochemistry. Histopathology.

[63] Kang B, Lee JM, Song YS, Woo S, Hur BY, Jeon JH, Paeng JC (2016). Added value of integrated whole-body PET/MRI for evaluation of colorectal cancer: comparison with contrast-enhanced MDCT. AJR Am J Roentgenol.

[64] Wolf AM, Fontham ET, Church TR (2018). Colorectal cancer screening for average-risk adults: 2018 guideline update from the American Cancer Society. CA Cancer J Clin.

[65] (2016). Recommendations on screening for colorectal cancer in primary care. CMAJ.

[66] Argilés G, Tabernero J, Labianca R (2020). Localised colon cancer: ESMO Clinical Practice Guidelines for diagnosis, treatment and follow-up. Ann Oncol.

[67] Saito Y, Oka S, Kawamura T (2021). Colonoscopy screening and surveillance guidelines. Dig Endosc.

[68] Chen FW, Sundaram V, Chew TA, Ladabaum U (2017). Advanced-stage colorectal cancer in persons younger than 50 years not associated with longer duration of symptoms or time to diagnosis. Clin Gastroenterol Hepatol.

[69] Marble K, Banerjee S, Greenwald L (1992). Colorectal carcinoma in young patients. J Surg Oncol.

[70] Siegel RL, Jakubowski CD, Fedewa SA, Davis A, Azad NS (2020). Colorectal cancer in the young: epidemiology, prevention, management. Am Soc Clin Oncol Educ Book.

[71] Kim TJ, Kim ER, Hong SN, Chang DK, Kim YH (2016). Long-term outcome and prognostic factors of sporadic colorectal cancer in young patients: a large institutional-based retrospective study. Medicine (Baltimore).

[72] Fu J, Yang J, Tan Y (2014). Young patients (≤ 35 years old) with colorectal cancer have worse outcomes due to more advanced disease: a 30-year retrospective review. Medicine (Baltimore).

[73] O'Connell JB, Maggard MA, Livingston EH, Yo CK (2004). Colorectal cancer in the young. Am J Surg.

[74] Chang DT, Pai RK, Rybicki LA (2012). Clinicopathologic and molecular features of sporadic early-onset colorectal adenocarcinoma: an adenocarcinoma with frequent signet ring cell differentiation, rectal and sigmoid involvement, and adverse morphologic features. Mod Pathol.

[75] Son IT, Kang JH, Kim BC, Park JH, Kim JW (2023). A retrospective multicenter study of the clinicopathological characteristics and prognosis of young adult patients with colorectal cancer: effects of chemotherapy on prognosis. J Clin Med.

[76] (2021). NCCN Clinical Practice Guidelines in Oncology: Colon Cancer. National Comprehensive Cancer Network. http:////efaidnbmnnnibpcajpcglclefindmkaj/https://www.nccn.org/professionals/physician_gls/pdf/colon.pdf.

[77] Amin MB, Greene FL, Edge SB (2017). The Eighth Edition AJCC Cancer Staging Manual: continuing to build a bridge from a population-based to a more "personalized" approach to cancer staging. CA Cancer J Clin.

[78] Yoshino T, Arnold D, Taniguchi H (2018). Pan-Asian adapted ESMO consensus guidelines for the management of patients with metastatic colorectal cancer: a JSMO-ESMO initiative endorsed by CSCO, KACO, MOS, SSO and TOS. Ann Oncol.

[79] Khan SA, Morris M, Idrees K (2016). Colorectal cancer in the very young: a comparative study of tumor markers, pathology and survival in early onset and adult onset patients. J Pediatr Surg.

[80] Bonin E, Bridoux V, Chati R, Kermiche S, Coget J, Tuech JJ, Roman H (2019). Diverting stoma-related complications following colorectal endometriosis surgery: a 163-patient cohort. Eur J Obstet Gynecol Reprod Biol.

[81] Son GM, Lee IY, Lee YS (2021). Is laparoscopic complete mesocolic excision and central vascular ligation really necessary for all patients with right-sided colon cancer?. Ann Coloproctol.

[82] Gosavi R, Chia C, Michael M, Heriot AG, Warrier SK, Kong JC (2021). Neoadjuvant chemotherapy in locally advanced colon cancer: a systematic review and meta-analysis. Int J Colorectal Dis.

[83] Shaw E, Warkentin MT, McGregor SE, Town S, Hilsden RJ, Brenner DR (2017). Intake of dietary fibre and lifetime non-steroidal anti-inflammatory drug (NSAID) use and the incidence of colorectal polyps in a population screened for colorectal cancer. J Epidemiol Community Health.

[84] Wakeman C, Keenan J, Eteuati J, Hollington P, Eglinton T, Frizelle F (2017). Chemoprevention of colorectal neoplasia. ANZ J Surg.

[85] Sáez J, Gimeno M, Cerrada E (2022). Synthesis of NSAID derivates for colon cancer targeted therapy.

[86] Simin J, Liu Q, Wang X (2021). Prediagnostic use of estrogen-only therapy is associated with improved colorectal cancer survival in menopausal women: a Swedish population-based cohort study. Acta Oncol.

[87] Mauri G, Pizzutilo EG, Amatu A (2019). Retreatment with anti-EGFR monoclonal antibodies in metastatic colorectal cancer: systematic review of different strategies. Cancer Treat Rev.

[88] Shitara K, Yamanaka T, Denda T (2019). REVERCE: a randomized phase II study of regorafenib followed by cetuximab versus the reverse sequence for previously treated metastatic colorectal cancer patients. Ann Oncol.

[89] Van Cutsem E, Köhne CH, Hitre E (2009). Cetuximab and chemotherapy as initial treatment for metastatic colorectal cancer. N Engl J Med.

[90] Bokemeyer C, Bondarenko I, Makhson A (2009). Fluorouracil, leucovorin, and oxaliplatin with and without cetuximab in the first-line treatment of metastatic colorectal cancer. J Clin Oncol.

[91] Golshani G, Zhang Y (2020). Advances in immunotherapy for colorectal cancer: a review. Therap Adv Gastroenterol.

[92] André T, Lonardi S, Wong KY (2022). Nivolumab plus low-dose ipilimumab in previously treated patients with microsatellite instability-high/mismatch repair-deficient metastatic colorectal cancer: 4-year follow-up from CheckMate 142. Ann Oncol.

[93] Merk C, Martling A, Lindberg J, Benhaim L, Taieb J, Lind P (2022). Circulating tumor DNA (ctDNA) in adjuvant therapy of early stage colon cancer: current status and future perspectives. Acta Oncol.

[94] Lieu CH, Golemis EA, Serebriiskii IG (2019). Comprehensive genomic landscapes in early and later onset colorectal cancer. Clin Cancer Res.

[95] Sanford NN, Giovannucci EL, Ahn C, Dee E, Mahal B (2020). Obesity and younger versus older onset colorectal cancer in the United States, 1998-2017. J Gastrointest Oncol.

[96] Zaki TA, Liang PS, May FP, Murphy CC (2023). Racial and ethnic disparities in early-onset colorectal cancer survival. Clin Gastroenterol Hepatol.

[97] D'Onofrio GM, Tan EG (1985). Is colorectal carcinoma in the young a more deadly disease?. Aust N Z J Surg.

[98] Ueno H, Ishiguro M, Nakatani E (2021). Prognostic value of desmoplastic reaction characterisation in stage II colon cancer: prospective validation in a Phase 3 study (SACURA Trial). Br J Cancer.

[99] Wang R, Wang MJ, Ping J (2015). Clinicopathological features and survival outcomes of colorectal cancer in young versus elderly: a population-based cohort study of SEER 9 Registries Data (1988-2011). Medicine (Baltimore).

[100] Abdelsattar ZM, Wong SL, Regenbogen SE, Jomaa DM, Hardiman KM, Hendren S (2016). Colorectal cancer outcomes and treatment patterns in patients too young for average-risk screening. Cancer.

[101] Siegel RL, Miller KD, Fedewa SA, Ahnen DJ, Meester RG, Barzi A, Jemal A (2017). Colorectal cancer statistics, 2017. CA Cancer J Clin.

[102] Sankila R, Aaltonen LA, Järvinen HJ, Mecklin JP (1996). Better survival rates in patients with MLH1-associated hereditary colorectal cancer. Gastroenterology.

